# Diagnostic Performance of Diffusion Tensor Imaging for Characterizing Breast Tumors: A Comprehensive Meta-Analysis

**DOI:** 10.3389/fonc.2019.01229

**Published:** 2019-11-18

**Authors:** Kai Wang, Zhipeng Li, Zhifeng Wu, Yucong Zheng, Sihui Zeng, Linning E, Jianye Liang

**Affiliations:** ^1^Department of Medical Imaging, Shanxi DAYI Hospital, Taiyuan, China; ^2^Department of Medical Imaging, State Key Laboratory of Oncology in South China, Collaborative Innovation Center for Cancer Medicine, Sun Yat-sen University Cancer Center, Guangzhou, China; ^3^Medical Imaging Center, The First Affiliated Hospital of Jinan University, Guangzhou, China

**Keywords:** diffusion tensor imaging, breast, standardized mean difference, diagnostic performance, magnetic resonance imaging, meta-analysis

## Abstract

**Rationale and Objectives:** Controversy still exists on the diagnosability of diffusion tensor imaging (DTI) for breast lesions characterization across published studies. The clinical guideline of DTI used in the breast has not been established. This meta-analysis aims to pool relevant evidences and evaluate the diagnostic performance of DTI in the differential diagnosis of malignant and benign breast lesions.

**Materials and Methods:** The studies that assessed the diagnostic performance of DTI parameters in the breast were searched in Embase, PubMed, and Cochrane Library between January 2010 and September 2019. Standardized mean differences and 95% confidence intervals of fractional anisotropy (FA), mean diffusivity (MD), and three diffusion eigenvalues (λ1, λ2, and λ3) were calculated using Review Manager 5.2. The pooled sensitivity, specificity, and area under the curve (AUC) were calculated with a bivariate model. Publication bias and heterogeneity between studies were also assessed using Stata 12.0.

**Results:** Sixteen eligible studies incorporating 1,636 patients were included. The standardized mean differences indicated that breast cancers had a significantly higher FA but lower MD, λ1, λ2, and λ3 than those of benign lesions (all *P* < 0.05). Subgroup analysis indicated that invasive breast carcinoma (IBC) had a significantly lower MD value than that of ductal carcinoma *in situ* (DCIS) (*P* = 0.02). λ1 showed the best diagnostic accuracy with pooled sensitivity, specificity, and AUC of 93%, 92%, and 0.97, followed by MD (AUC = 0.92, sensitivity = 87%, specificity = 83%) and FA (AUC = 0.76, sensitivity = 70%, specificity = 70%) in the differential diagnosis of breast lesions.

**Conclusion:** DTI with multiple quantitative parameters was adequate to differentiate breast cancers from benign lesions based on their biological characteristics. MD can further distinguish IBC from DCIS. The parameters, especially λ1 and MD, should attract our attention in clinical practice.

## Introduction

Breast cancer is the most commonly diagnosed cancer and the leading cause of cancer death among females in the world based on the GLOBOCAN 2018 estimates of cancer incidence and mortality (1). Early detection and accurately discriminating breast cancer from benign lesions play an important role in the determination of therapeutic regimen, which may help improve the disease-free survival and overall survival when the patients were diagnosed early and timely treated.

Breast MRI is the most sensitive imaging tool for breast cancer detection and shows superiority in the dense breast with rich glands (2). However, the specificities of conventional sequences are modest even combined with dynamic contrast-enhanced (DCE) MRI (3). This uncertainty may lead to unnecessary biopsies.

In recent years, diffusion tensor imaging (DTI), an extension of diffusion weighted imaging (DWI), has been used to characterize breast lesions and shows promising results in increasing diagnostic specificity (4). It can calculate the anisotropy and directionality of water diffusion in tissues by encoding the diffusion in six or more directions (5). The DTI parameters including fractional anisotropy (FA), mean diffusivity (MD), and three orthogonal diffusion coefficients (λ1, λ2, λ3) can provide subtle information regarding microstructure and pathophysiology of the breast, which help distinguish different lesions. Several studies indicated that DWI-derived apparent diffusion coefficient (ADC) values, also named MD in DTI protocol, significantly decreased in breast cancers compared with benign lesions, and it also increased the ability of DCE-MRI to differentiate cancers from benign lesions (4, 6). However, whether DTI-derived parameters have comparable diagnostic accuracy to DWI in a large cohort of patients is still unclear. Besides, there are still some controversies in the differentiation of breast lesions using DTI among published studies. For instance, most studies (5, 7, 8) showed that breast cancers have higher FA and lower MD, λ1, λ2, and λ3 values than those of benign lesions while Partridge et al. (9), Cakir et al. (10), and Eyal et al. (11) reported that there was no statistical difference in FA between malignant and benign lesions. MD but not FA, volume ratio, and relative anisotropy values can further distinguish invasive breast carcinoma (IBC) from ductal carcinoma *in situ* (DCIS) in the study of Wang et al. (12), which decreased the diagnostic confidence of DTI to a certain extent. Last, the most sensitive parameters among DTI in characterizing breast lesions were not completely consistent between studies. Therefore, we summarized previously published results regarding the diagnostic performance of DTI parameters in differentiating breast cancer from benign lesions with a meta-analysis method. The pooled outcomes may address the controversial findings between different studies and provide more reliable information to clinicians.

## Materials and Methods

### Data Sources

Two of the authors searched for any literature regarding differentiation between breast cancer and benign lesions using DTI in Embase, PubMed, and Cochrane Library between January 2010 and September 2019. The formula consisted of (breast cancer or carcinoma or malignance), (DTI, diffusion tensor imaging) and (diagnostic performance or differentiation), with the searching limitations in the title or abstract. We also searched relevant references from included studies and performed manual retrieval if necessary.

### Study Selection

The inclusion criteria were as follows: (a) DTI was used to differentiate breast cancer from benign lesions; (b) sufficient data regarding mean and standard deviation (SD) or diagnostic performance of DTI parameters [i.e., sensitivity, specificity, true-positive (TP), false-negative (FN), false-positive (FP), and true-negative (TN)] were reported or can be calculated from the study; (c) the breast lesions were confirmed by pathology; (d) the patients have not been treated with surgery or chemotherapy before magnetic resonance scanning; and (e) the scores of quality assessment based on likelihood of bias were at least 9. The exclusion criteria were listed as follows: (a) case report, review, letter to editor, meta-analysis, or conference abstract; (b) preclinical studies; (c) not a breast study or primary breast tumors; (d) without sufficient data or lack of comparisons.

### Data Abstraction and Quality Assessment

Two authors extracted the basic information from each study, which included first author, publication year, country, machine type, b values, number of imaging directions, age of patients, tumor diameters, and publication journal. The following data were also extracted for calculating the pooled effects: mean value and standard deviation of DTI parameters, TP, FN, FP, TN, sensitivity, specificity, threshold values, and area under the curve (AUC). If the sensitivity and specificity are not provided, we will extract them from the receiver operating characteristic curves. Once the numbers of benign and malignant lesions, sensitivity, and specificity are known, we can recalculate TP, FN, FP, and TN using the calculator from Review Manager 5.2 for further pooling. The Revised Quality Assessment of Diagnostic Accuracy Studies (QUADAS-2) checklist was applied to assess the quality of included studies, with 14 criteria based on the risk of bias (13). The criteria were judged as “Yes (low risk of bias),” “No (high risk of bias),” or “Unclear.” We discussed or invited a statistician to achieve a consensus when the results were controversial.

### Data Synthesis

The pooled effects and 95% confidence intervals (CIs) for each parameter were calculated using Review Manager Software version 5.3 (Cochrane Collaboration, Oxford, UK). The Begg test was used to evaluate the publication bias for the continuous variables (i.e., mean values and standard deviations) while the Deek plots evaluated the publication bias regarding diagnostic test with sensitivity and specificity using Stata version 12.0 (StataCorp LP, College Station, TX). Asymmetric or skewed funnel plots indicated the potential of publication bias. *P* < 0.05 of Begg's test indicated the presence of publication bias. Inconsistency index (*I*^2^) and Cochran's *Q*-tests were applied to evaluate the degree of heterogeneity between studies, which may originate from selected b-values, number of imaging directions, tumor subtypes, and so on. *I*^2^ > 50% or *P* < 0.05 for Cochran *Q*-test indicated the presence of heterogeneity, and a random-effects model was used to calculate the pooled results. Otherwise, a fixed-effect model was applied. As the parameters varied to some extent among included studies, standardized mean difference (SMD) was used to pool the continuous variables, which suggested less heterogeneity compared with weighted mean difference (14). Stata.12.0 was applied to calculate the pooled sensitivity, specificity, positive likelihood ratio, negative likelihood ratio, diagnostic odds ratio, AUC, and their 95% CIs with a bivariate mixed-effects binary regression model. The receiver operating characteristic curve was used to determine the diagnostic values of FA, MD, and λ1 in the differentiation between breast cancer and benign lesions (15). Fagan nomograms were plotted to predict posttest probability of FA, MD, and λ1 for the diagnosis of breast cancer.

## Results

### Literature Search and Selection of Studies

**Figure 1:**
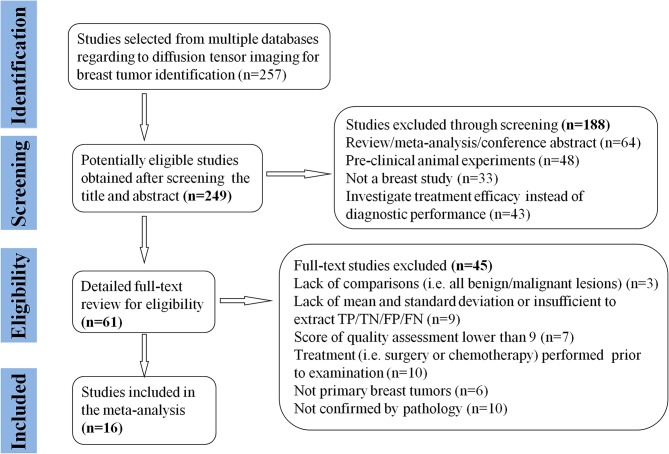
Flowchart of selection and exclusion process. Sixteen studies that met the inclusion criteria are eventually included. FN, false negative; FP, false positive; TN, true negative; TP, true positive.

A total of 257 potential studies were obtained after searching the keywords in titles and abstracts from multiple databases. We excluded 188 studies after a review of the titles and abstracts, which consisted of reviews, meta-analysis, conference abstracts, and preclinical animal studies. Some studies were excluded for not a breast or diagnostic study. We downloaded and read the full texts of the remaining 61 studies and excluded an additional 12 studies because of lack of comparisons or sufficient data. The studies with low-quality scores that indicated high risk of bias, treatment performed prior to examination, and the tumors that did not originate from the breast or had not been confirmed by pathology led to exclude 33 studies. Finally, a total of 16 studies comprising 927 malignant and 709 benign lesions were included in the meta-analysis. The breast cancers mainly consisted of invasive ductal carcinoma, invasive lobular carcinoma, DCIS, papillary carcinoma, mucinous carcinoma, medullary carcinoma, and mixed types. The IBC included all invasive subtypes of breast cancers except DCIS. The benign lesions mainly consisted of fibroadenomas, sclerosing adenosis, fibrosis foci, phyllodes tumors, chronic inflammations, and normal fibroglandular tissue. A flowchart detailing the selection process based on inclusion and exclusion criteria is shown in Figure 1. Basic characteristics and diagnostic performance of included studies are summarized in Tables 1, 2, respectively.

**Table 1:** Basic characteristics of studies included in the meta-analysis.

**References**	**Year**	**Country**	**Malignant (*n*)**	**Benign(n)**	**Machine type**	**b- values (s/mm^**2**^)**	**Gradient directions**	**Age (years)**	**Tumor diameters (mm)**	**Journal**	**Quality assessment**
Luo et al. (16)	2019	USA	95	143	3T Philips	0, 100, 800	6	51 (23–83)	11 (4–114)	Breast Cancer Research	13
Si et al. (17)	2016	China	35	39	3T Siemens	0, 800	20	46 (24–74)	>10	Natl Med J China	9
Jiang et al. (18)	2016	China	34	22	1.5T Siemens	0, 1000	6	47 (37–68)	>10	Med Sci Monit	11
Furman-Haran et al. (19)	2016	Israel	24	6	3T Siemens	0, 700	20	51 (38–72)	24 ± 13	J Magn Reson Imaging	13
Cakir et al. (10)	2013	Turkey	30	25	3T Philips	0, 1,000	16	45.1 (21–73)	24.0 ± 9.76	Eur J Radiol	12
Onaygil et al. (5)	2017	Germany	45	47	3T Siemens	0, 700	30	Benign: 37.9 ± 10.8 Malignant: 51.8 ± 13.6	>10	J Magn Reson Imaging	10
Yamaguchi et al. (20)	2016	Japan	58	22	1.5T Siemens	0, 1,000	6	Benign: 46 Malignant: 62.5	Benign: 19.5 (6–90); Malignant: 15 (10–60)	Magn Reson Med Sci	9
Tsougos et al. (21)	2018	Greece	42	44	3T GE	0, 600	6	53 ± 13	Benign: 17 (6–51); Malignant: 28 (7–90)	Clin Imaging	12
Ozal et al. (22)	2018	Turkey	46	46	3T Siemens	0, 1,000	6	55.88 ± 10.92	29.26 ± 10.09	Niger J Clin Pract	11
Abdel Razek et al. (23)	2019	Egypt	13	17	1.5T Philips	0, 1,000	12	27–58	NA	Eur J Radiol	13
Kim et al. (24)	2018	Korea	251	251	3T Siemens	0, 1,000	20	53.8 (25–83)	24 (5–95)	Eur Radiol	13
Baltzer et al. (7)	2011	Germany	54	17	1.5T Siemens	0, 1,000	6	54.6 ± 15.7	>5	Eur Radiol	9
Partridge et al. (9)	2010	USA	76	29	1.5T GE	0, 600	6	53 (22–85)	NA	J Magn Reson Imaging	11
Teruel et al. (8)	2016	Norway	38	34	3T Siemens	0, 700	30	46 (17–79)	NA	J Magn Reson Imaging	10
Wang et al. (12)	2015	China	53	0	1.5T GE	0, 600	6	50.11 ± 10.09	NA	Chin J Cancer Res	12
Eyal et al. (11)	2012	Israel	33	20	3T Siemens	0, 700	30	Benign: 40 (26–65) Malignant: 52 (31–78)	Benign: 12 (10–18); Malignant: 20 (14–27)	Invest Radiol	11

*NA, not available*.

**Table 2:** Detailed information regarding diagnostic performance in each study.

**Parameters**	**References**	**Year**	**Sensitivity**	**Specificity**	**AUC**	**TP**	**FP**	**FN**	**TN**	**Threshold**
FA	Si et al. (17)	2016	0.462	0.857	0.638	16	6	19	33	0.207
	Jiang et al. (18)	2016	0.441	0.773	0.607	15	5	19	17	0.189
	Onaygil et al. (5)	2017	0.644	0.766	0.760	29	11	16	36	0.170
	Tsougos et al. (21)	2018	0.729	0.658	0.729	31	15	11	29	NA
	Abdel Razek et al. (23)	2019	0.923	0.706	0.820	12	5	1	12	0.470
	Baltzer et al. (7)	2011	0.796	0.647	0.770	43	6	11	11	0.194
	Partridge et al. (9)	2010	0.650	0.279	0.500	49	21	27	8	0.240
	Teruel et al. (8)	2016	0.868	0.824	0.896	33	6	5	28	0.104
MD	**Luo et al**. **(****16****)**	**2019**	**0.738**	**0.642**	**0.750**	**70**	**51**	**25**	**92**	**NA**
	**Si et al**. **(****17****)**	**2016**	**0.846**	**0.914**	**0.944**	**30**	**3**	**5**	**36**	**1.370**
	**Jiang et al**. **(****18****)**	**2016**	**0.824**	**0.909**	**0.897**	**28**	**2**	**6**	**20**	**1.017**
	**Cakir et al**. **(****10****)**	**2013**	**1.000**	**0.400**	**0.820**	**30**	**15**	**0**	**10**	**1.270**
	**Yamaguchi et al**. **(****20****)**	**2016**	**0.910**	**0.860**	**0.924**	**53**	**3**	**5**	**19**	**1.338**
	**Tsougos et al**. **(****21****)**	**2018**	**0.825**	**0.814**	**0.906**	**35**	**8**	**7**	**36**	**NA**
	**Baltzer et al**. **(****7****)**	**2011**	**0.870**	**0.882**	**0.894**	**47**	**2**	**7**	**15**	**1.160**
	**Partridge et al**. **(****9****)**	**2010**	**0.732**	**0.583**	**0.760**	**56**	**12**	**20**	**17**	**1.200**
	**Teruel et al**. **(****8****)**	**2016**	**0.941**	**0.947**	**0.968**	**36**	**2**	**2**	**32**	**1.330**
	**Onaygil et al**. **(****5****)**	**2017**	**0.956**	**0.936**	**0.969**	**43**	**3**	**2**	**44**	**1.240**
	**Abdel Razek et al**. **(****23****)**	**2019**	**0.769**	**0.824**	**0.860**	**10**	**3**	**3**	**14**	**1.100**
λ1	Si et al. (17)	2016	1.000	0.971	0.987	35	1	0	38	1.393
	Jiang et al. (18)	2016	0.853	0.909	0.898	29	2	5	20	1.220
	Onaygil et al. (5)	2017	0.978	0.872	0.950	44	6	1	41	1.590
	Tsougos et al. (21)	2018	0.825	0.814	0.906	35	8	7	36	NA
	Teruel et al. (8)	2016	0.912	0.974	0.961	35	1	3	33	1.570
	Eyal et al. (11)	2012	0.956	0.977	0.994	32	0	1	20	1.500

*NA, not available; FA, fractional anisotropy; λ1, prime diffusion coefficient; MD, mean diffusivity; AUC, area under the curve; FN, false negative, FP, false positive; TN, true negative, TP, true positive. Threshold values of λ1 and MD are factors of 10^−3^ mm^2^/s*.

### Quantitative Analysis

#### FA for Differentiation of Breast Lesions

**Figure 2:**
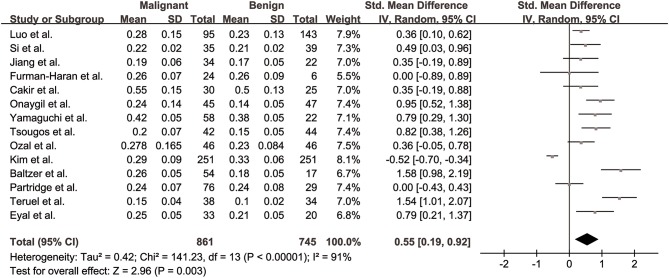
Forest plot of the mean value and standard deviation of fractional anisotropy (FA) between breast cancer and benign lesions. The standardized mean differences indicated that breast cancers had a significant higher FA than benign lesions.

**Figure 3:**
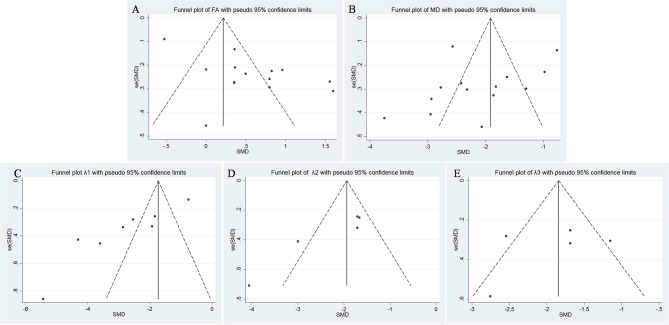
Funnel plot of **(A)** fractional anisotropy (FA), **(B)** mean diffusivity (MD), **(C)** λ1, **(D)** λ2, and **(E)** λ3. Only λ1 showed potential publication bias.

FA values of breast cancer and benign lesions from 14 studies were compared. Heterogeneity tests showed χ^2^ = 141.23, *I*^2^ = 91%, *P* < 0.001, indicating obvious heterogeneity between studies. The forest plot of the mean value and standard deviation of FA between breast cancer and benign lesions was shown in Figure 2. The SMD of FA value was pooled using a random-effects model, and the result was 0.55 (0.19, 0.92), *P* = 0.003. The funnel plot was symmetric overall (Figure 3), and no obvious publication bias was observed using Begg test (*P* = 0.511).

#### MD for Differentiation of Breast Lesions

**Figure 4:**
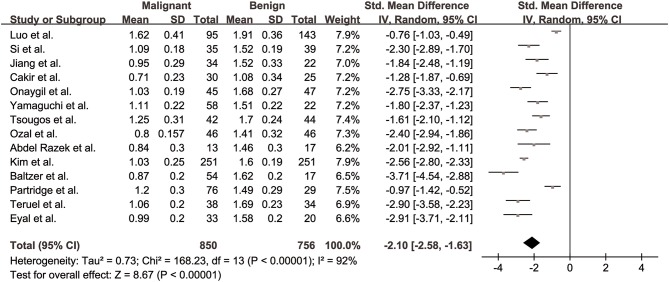
Forest plot of the mean value and standard deviation of mean diffusivity (MD) between breast cancer and benign lesions. The standardized mean differences indicated that breast cancers had a significantly lower MD than benign lesions.

MDs of breast cancer and benign lesions from 14 studies were compared. Heterogeneity tests showed χ^2^ = 168.23, *I*^2^ = 92%, *P* < 0.001, indicating obvious heterogeneity between studies. The forest plot of the mean value and standard deviation of MD between breast cancer and benign lesions was shown in Figure 4. The SMD of MD value was pooled using a random-effects model, and the result was −2.10 (−2.58, −1.63), *P* < 0.001. The funnel plot was symmetric overall (Figure 3), and no obvious publication bias was observed using Begg test (*P* = 0.125).

#### Prime Diffusion Eigenvalue (λ1) for Differentiation of Breast Lesions

**Figure 5:**
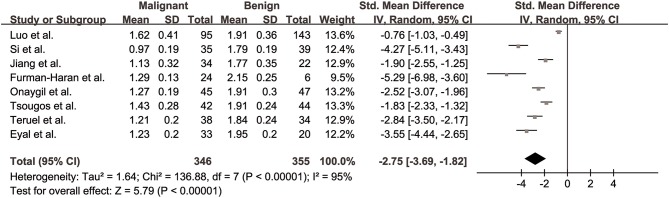
Forest plot of the mean value and standard deviation of prime diffusion eigenvalue (λ1) between breast cancer and benign lesions. The standardized mean differences indicated that breast cancers had a significant lower λ1 than benign lesions.

The λ1 values of breast cancer and benign lesions from eight studies were compared. Heterogeneity tests showed χ^2^ = 136.88, *I*^2^ = 95%, *P* < 0.001, indicating obvious heterogeneity between studies. The forest plot of the mean value and standard deviation of λ1 between breast cancer and benign lesions was shown in Figure 5. The SMD of λ1 was pooled using a random-effects model, and the result was −2.75 (−3.69, −1.82), *P* < 0.001. The funnel plot was asymmetric, which lacked negative studies at the right bottom (Figure 3). *P* = 0.009 of Begg test suggested significant publication bias.

#### λ2 for Differentiation of Breast Lesions

**Figure 6:**
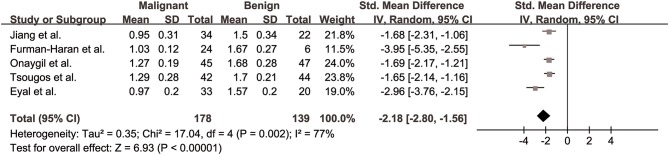
Forest plot of the mean value and standard deviation of λ2 between breast cancer and benign lesions. The standardized mean differences indicated that breast cancers had a significant lower λ2 than benign lesions.

The λ2 values of breast cancer and benign lesions from five studies were compared. Heterogeneity tests showed χ^2^ = 17.04, *I*^2^ = 77%, *P* = 0.002, indicating moderate heterogeneity between studies. The forest plot of the mean value and standard deviation of λ2 between breast cancer and benign lesions was shown in Figure 6. The SMD of λ2 was pooled using a random-effects model, and the result was −2.18 (−2.80, −1.56), *P* < 0.001. The funnel plot was symmetric overall (Figure 3). *P* = 0.086 of Begg test suggested no publication bias.

#### λ3 for Differentiation of Breast Lesions

**Figure 7:**
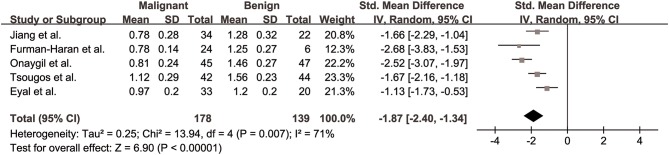
Forest plot of the mean value and standard deviation of λ3 between breast cancer and benign lesions. The standardized mean differences indicated that breast cancers had a significant lower λ3 than benign lesions.

The λ3 values of breast cancer and benign lesions from five studies were compared. Heterogeneity tests showed χ^2^ = 13.94, *I*^2^ = 71%, *P* = 0.007, indicating moderate heterogeneity between studies. The forest plot of the mean value and standard deviation of λ3 between breast cancer and benign lesions was shown in Figure 7. The SMD of λ3 was pooled using a random-effects model, and the result was −1.87 (−2.40, −1.34), *P* < 0.001. The funnel plot was symmetric overall (Figure 3). *P* = 0.806 of Begg test suggested no publication bias.

#### Subgroup Analysis of MD and FA for Differentiation Between IBC and DCIS

Three studies for MD (9, 12, 18) and four studies for FA values (9, 12, 18, 24) used in the differentiation between two subtypes of breast cancer were further pooled. The SMD of MD was −0.76 (−1.40, −0.12), *P* = 0.02, which indicated that IBC had a lower MD value than that of DCIS. *I*^2^ = 64% suggested mild heterogeneity. However, no significant difference was observed in FA value with an SMD of 0.16 (−0.12, 0.45), *P* = 0.26, indicating FA cannot further distinguish IBC from DCIS. More studies were necessary to draw a reliable conclusion in the future.

### Diagnostic Performance of DTI Parameters

**Figure 8:**
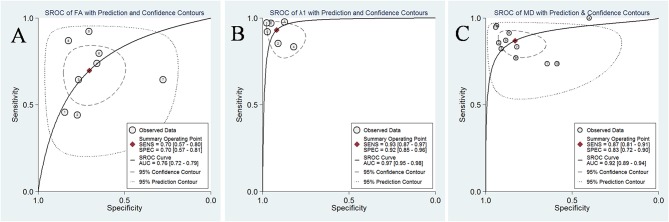
Summary receiver operating characteristic (SROC) curve of **(A)** fractional anisotropy (FA), **(B)** λ1, and **(C)** mean diffusivity (MD) in the discrimination of breast lesions. λ1 showed the largest area under the curve among the three parameters, followed by MD and FA.

**Figure 9:**
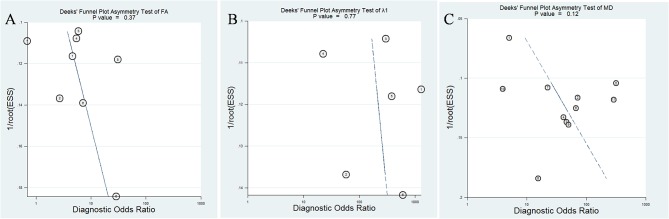
Deeks' funnel plot for **(A)** fractional anisotropy (FA), **(B)** λ1, and **(C)** mean diffusivity (MD). No publication bias was indicated in the three parameters.

The pooled sensitivity, specificity, positive likelihood ratios (PLRs), negative likelihood ratios (NLRs), diagnostic odds ratios, and AUCs of FA, λ1, and MD were listed in Table 3. The summary receiver operating characteristic curves are shown in Figure 8. No significant publication bias was observed in Deeks' plots for the three parameters (Figure 9). λ1 showed the highest AUC value of 0.97, with the sensitivity and specificity of 0.93 and 0.92, followed by MD (AUC = 0.92, sensitivity = 87%, specificity = 83%) and FA (AUC = 0.76, sensitivity = 70%, specificity = 70%) in the differential diagnosis of breast lesions.

**Table 3:** Pooled estimates and heterogeneity measures for FA, λ1, and MD.

	**Sensitivity**	**Specificity**	**PLR**	**NLR**	**DOR**	**AUC**	***I***^****2****^	
							**Sensitivity (%)**	**Specificity (%)**
FA	0.70 (0.57, 0.80)	0.70 (0.57, 0.81)	2.4 (1.5, 3.6)	0.43 (0.29, 0.64)	5 (3, 12)	0.76 (0.72, 0.79)	75.63	76.34
λ1	0.93 (0.87, 0.97)	0.92 (0.85, 0.96)	11.3 (5.9, 21.7)	0.07 (0.04, 0.15)	151 (47, 489)	0.97 (0.95, 0.98)	60.10	52.37
MD	0.87 (0.81, 0.91)	0.83 (0.72, 0.90)	5.1 (2.9, 8.8)	0.16 (0.10, 0.24)	32 (13, 79)	0.92 (0.89, 0.94)	76.08	87.20

*The data in parentheses indicate mean and 95% confidence intervals. FA, fractional anisotropy; λ1, prime diffusion coefficient; MD, mean diffusivity; PLR, positive likelihood ratio; NLR, negative likelihood ratio; DOR, diagnostic odds ratio; AUC, area under the curve; I^2^, inconsistency index*.

**Figure 10:**
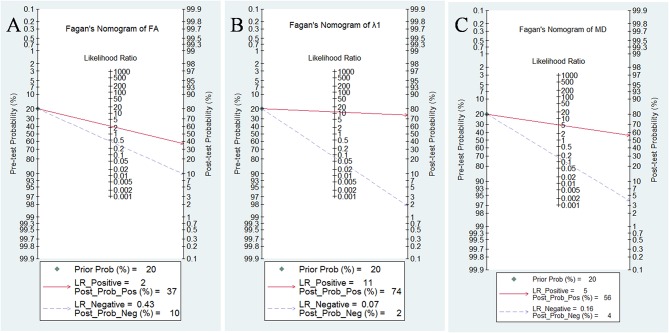
Fagan's nomogram of **(A)** fractional anisotropy (FA), **(B)** λ1, and **(C)** mean diffusivity (MD).

Likelihood ratios and posttest probabilities are also relevant for clinicians (25, 26). They provide information about the likelihood that a patient is diagnosed with breast cancer or not under certain parameters. Fagan's nomograms of FA, λ1, and MD were shown in Figure 10. We set all of the pretest probabilities at 20%. Using FA value would raise the posttest probability to 37% when pretest positive from 20% with a PLR of 2.4 and would reduce the posttest probability as low as 10% when negative with an NLR of 0.43. In our study, diagnosing breast cancer is regarded as a positive event and corresponds to a higher FA, while diagnosing benign lesion is regarded as a negative event and corresponds to a lower FA. This suggests that the diagnostic tendency of breast cancer will significantly increase to 37% with the help of FA value (a higher FA) compared with the condition without the prompt of FA value whose diagnostic probability was set at 20% beforehand. On the contrary, the lower the posttest probability is when a negative event occurs (the DTI shows a lower FA), the higher probability for diagnosing benign lesions will be. Similarly, the posttest probability of MD reached 56% with a PLR of 5.1 and would reduce the posttest probability as low as 4% when negative with an NLR of 0.16. The posttest probability of λ1 reached 74% with a PLR of 11.3 and would reduce the posttest probability as low as 2% when negative with an NLR of 0.16, indicating that it has the best predictive ability to diagnose breast cancer or benign lesions depending on its value. The results suggested that DTI parameters were helpful for increasing the accuracy in detecting breast cancer and also indicated that λ1 was the most valuable parameter in the characterization of breast lesions.

## Discussion

DTI characterizes tissue microstructure and water diffusion directionality by performing diffusion sensitization in multiple orientations (27). It can evaluate tumor invasiveness of glioblastoma, and the fiber-tracking based neuronavigation has been successfully used in preoperative surgical planning (28–30). However, the clinical guideline of DTI used in the breast has not been established. Therefore, we performed a meta-analysis to pool relevant evidences that assessed the diagnostic accuracy of DTI for breast lesion detection. This study showed reliable results and promising prospects for clinical application of DTI in the breast.

In this meta-analysis, the SMD indicated that breast cancers had a higher FA but lower MD, λ1, λ2, and λ3 than those of benign lesions. MD and FA in breast lesions had been reported to be significantly correlated to tissue cellularity, and breast cancer had a higher cellularity than benign lesions (31). The observed reduction of diffusion coefficients may result from the higher cellularity of cancerous tissues, which would restrict the diffusion activity of water molecules in the extracellular compartment. Besides, blockage of the ducts and lobules by cancer cells may also contribute to the decrease of diffusion coefficients in all directions (19). FA reflects the degree of preferred directionality to water motion and can show the microstructures and arrangements of tissues. Previous study demonstrated a positive correlation between FA and tumor cell density in glioblastomas (32). Besides, complicated and disordered structures with regional hemorrhage or liquefactive necrosis were more easily seen in cancerous tissues, which may enhance the diffusion of water molecules in certain directions while reduced in others in the more disordered microstructures of cancerous tissues (23). This partly explained the increase of anisotropy in breast cancer. In the study of Luo et al. (16), they reported that higher FA was associated with malignancy for masses and benign non-masses, which suggested that DTI anisotropy metrics must be considered in the context with lesion type for diagnostic purposes. Theoretically, water diffusion in the fibrous connective tissues, glandular tissues, and cysts, which are major components of benign lesions in the breast, is close to isotropic (19). It is worth noting that a larger area of liquefactive necrosis more easily occurs in a highly aggressive and large tumor, which may result in a loss of organization of the breast parenchyma in cancerous tissues, and in reverse reduce the anisotropy to a certain extent in breast cancer, as well as the difference of FA between breast cancer and benign lesions. It may be the reason that FA reported in some of the included studies cannot distinguish breast cancer from benign lesions (9, 18, 19). FA also performed a moderate diagnostic value, with the lowest AUC of 0.76 among the three parameters. In our study, λ1 showed a higher AUC than FA and MD in the differentiation of breast lesions. Fagan's nomograms also suggested that λ1 had a higher posttest probability with a PLR of 11.3 and a lower posttest probability when negative with an NLR of 0.16. The measurement of MD is non-directional and unable to reveal three-dimensional diffusion mobility, which may result from the disordered structure. In normal breast tissue, the ducts, vessels, and other parenchyma around them are arranged in a relatively parallel pattern and have their main diffused direction. As a result, the water molecule moves mainly along the primary axis in the extracellular space and demonstrates anisotropy. As a number of diffusion gradients were applied in DTI, it can uniquely determine a specific three-dimensional ellipsoid tensor unit in each pixel, within which the diffusion ability in any direction can be accurately calculated (18). Therefore, DTI-derived λ1, which were calculated from three-dimensional tissue volumes, displayed superior diagnostic performance compared to MD. Most importantly, DTI-derived parameters showed a much higher specificity (as high as 92%) in detecting breast cancer compared to DCE-MRI alone, whose specificity was reported to be only 71% in a previous meta-analysis (6).

In clinical practice, systemic treatments such as chemotherapy, biotherapy, and radiotherapy are needed in addition to surgery to control local recurrence and distant metastasis for most patients with IBC. Inspiringly, the pooled results suggested that MD can further distinguish IBC from DCIS. Wang et al. (12) reported that invasive carcinomas had a higher cellular density and more crowded extracellular matrix than DCIS that inhibited water movement. Besides, interstitial fibrosis as a result of a desmoplastic reaction was observed in the stroma of IBC, which led to a decrease of MD in IBC. However, FA failed to identify the subtle difference between them. In the study of Jiang et al. (18), they found that DCIS has great variation in tumor grade and cellularity, and some high-grade DCIS has relatively higher cellularity, which may decrease the specificity of FA to a certain extent. In individual studies, MD or FA showed significant correlations with the ER status, Ki67 labeling index, and nuclear/histological grade and could detect lymphovascular invasion and axillary node metastasis in patients with breast cancer (5, 20, 24). However, the results were not completely consistent, and further researches with a large cohort of patients are necessary.

Exploration of heterogeneity was an indispensable part of meta-analysis when analyzing the pooled results. Introducing improper heterogeneity will decrease the credibility of the findings. Although we set strict selection criteria for including high-quality studies, FA, λ1, λ2, λ3, and MD still demonstrate moderate to obvious heterogeneity (*I*^2^ ranged from 71 to 92%). Several potential confounding factors should be noticed. First, both 1.5T and 3.0T MR scanners were used to image the breast lesions in the included studies. Higher field strength will provide clear images with higher signal-noise ratio to delineate the lesions. Second, the b-values selected and numbers of gradient directions varied from study to study, which may influence the calculation of DTI parameters. Third, only a rough comparison was performed between breast cancer and benign lesions. Their compositions, hormone status, invasiveness, tumor subtypes, lesion sizes, and type of genetic mutations may form a completely different biologic behavior and structural characteristic that confounded the results. Last, we included both negative and positive results for this meta-analysis, which would introduce reasonable heterogeneity, but the publication bias was reduced.

There are some limitations in this study. First, other meaningful DTI parameters such as maximal anisotropy, relative anisotropy, volume ratio, geodesic anisotropy, average eigenvalues, and radial diffusion have not been pooled due to limited number of studies. Second, intravoxel-incoherent-motion DWI, which can reflect the microcirculation perfusion of the lesions, is also an important sequence for breast imaging. The diagnostic performance between this sequence and DTI has not been compared. Third, the studies and sample sizes vary between FA, λ1, and MD comparisons; confounding may be an issue as the studies are likely to be heterogeneous. Last, publication bias was found in λ1 value, but we directly pooled the results instead of using the trim and fill method (33).

In conclusion, breast cancers showed a significantly higher FA but lower MD, λ1, λ2, and λ3 compared to benign lesions. DTI is a valuable tool to differentiate breast cancer from benign lesions with high sensitivity and specificity. Its parameters can add specificity to the detection of breast cancer compared with DCE-MRI. MD showed potential to distinguish IBC from DCIS. There are still controversies in the explanation of FA for the difference between breast cancer and benign lesions, and we should pay caution to its usage. The parameters, especially λ1 and MD, should attract our attention in clinical practice. Besides, the applications of DTI in reflecting ER status, Ki67 status, tumor invasiveness, and the relations with lymphovascular invasion and axillary node metastasis are promising research directions.

## Data Availability Statement

All data sets analyzed for this study are included in the article/supplementary material.

## Ethics Statement

The present study was not a primary research involving human samples in the public databases.

## Author Contributions

JL, SZ, and LE contributed to the conception and design of this research. KW and ZL contributed to the drafting of the article and final approval of the submitted version. ZW and YZ contributed to data analyses and the interpretation and completion of the figures and tables. All authors read and approved the final manuscript.

### Conflict of Interest

The authors declare that the research was conducted in the absence of any commercial or financial relationships that could be construed as a potential conflict of interest.

## Abbreviations

DTI: diffusion tensor imaging
DWI: diffusion weighted imaging
IBC: invasive breast carcinoma
DCIS: ductal carcinoma *in situ*
FA: fractional anisotropy
λ1-3: eigenvalue
MD: mean diffusivity
FN: false negative
FP: false positive
TN, true negative, TP: true positive
PLR: positive likelihood ratio
NLR: negative likelihood ratio
DOR: diagnostic odds ratio
AUC: area under the curve
*I*^2^: inconsistency index
CI: confidence interval
SMD: standardized mean difference.
